# A photovoice study of school belongingness among high school students in Norway

**DOI:** 10.1080/22423982.2017.1421369

**Published:** 2018-01-02

**Authors:** Vaiva Sunniva Deraas Lieblein, Maria Warne, Suzanne Huot, Debbie Laliberte Rudman, Ruth Kjærsti Raanaas

**Affiliations:** ^a^ Department of Public Health Science, Faculty of Landscape and Society, Norwegian University of Life Sciences, Ås, Norway; ^b^ Department of Health Sciences, Mid Sweden University, Östersund, Sweden; ^c^ Department of Occupational Science and Occupational Therapy, University of British Columbia, Vancouver, Canada; ^d^ School of Occupational Therapy, University of Western Ontario, London, Canada

**Keywords:** Upper secondary school, school dropout, high north, youth, health promotion

## Abstract

Although high school graduation is important for living conditions and health throughout life, many students do not complete. In Norway’s northern most county, Finnmark, up to 45% of students do not complete high school. Contrary to prior research that has primarily focused on causes for dropout, this study’s aim was to deepen understanding of factors that support high school attendance. A strengths-based participatory approach using photovoice addressed attendance factors as perceived by seven participating students from one high school in Finnmark. Qualitative content analysis of data generated through group dialogue about participant-generated photos and individual interviews identified six factors important for students’ school attendance: a supportive school environment, a good learning environment, recuperation and recreation, family and friends, goals and ambitions, and place attachment. Related aspects of a supportive environment and belongingness, where school staff made important contributions to promoting a positive environment, were essential.

## Introduction

Graduation from high school is important for health and living conditions, with dropout linked to health and social problems later in life [1,2]. Relatedly, education is a primary social determinant of health, with duration and quality of life increasing with increased length of education [3].

Despite the many challenges associated with school dropout, approximately 27% of students in Norway do not graduate from high school (defined as not completing within 5 years of initiation), 33% for males and 22% for females [4]. In Norway, high school refers to 3 years of optional school that follow 10 years of mandatory school. The students can choose from a number of different programmes, both theoretical and vocational. Dropout rates from theoretical programmes are 14% and 41% for vocational programmes [4]. The proportion of dropout varies between regions. In Finnmark, the northern most county in Norway, the share is as high as 45%, 54% for males and 36% for females.

In turn, governmentally supported programmes and reforms have been developed to prevent dropout, such as No Child Left Behind in the USA and the NyGIV-project in Norway [5]. Further knowledge development addressing preventative actions, that considers contextual factors, can optimise strategies to reduce rates. This study focuses on Finnmark given its high student dropout rates.

To date, research and policy have typically attributed failure to complete high school to student or family characteristics, with an emphasis on students’ insufficient level of engagement [6,7], inappropriate behaviour [8] or lack of understanding of educational benefits. Families have been framed as insufficient role models, or blamed for not being supportive of education [9,10]. In Finnmark, many role models do not have higher education [11] due to the fact that primary industries such as fishing and reindeer herding have dominated until recently.

Although some studies on school dropout have focused on school quality [e.g. 12] and community factors, few studies have examined wider economic, political and social factors [13]. For example, Finnmark covers the largest land area (48,618 km^2^) of all counties in Norway, but has only 10 high schools given low population density. Due to large distances and limited public transport, many high school students live away from home to attend school, staying in dormitories or renting rooms privately. Thus, many students need to be able to take care of themselves from the age of 15–16 years, and being overwhelmed with this responsibility can contribute to drop out [11]. Because of the long distances and often limited opportunities to choose between study programmes at the different schools, some students select their second or third study programme priority to enable living closer to their home, family and friends. Leaving out their first priority choice may also contribute to dropout [11].

One relevant concept in furthering understanding of school dropout is place belongingness, or sense of belongingness to school. Place belongingness refers to an individual’s feeling of being “at home” in the symbolic sense of being familiar, comfortable, secure and emotionally attached to a place, a culture, a group or even a situation [14,15]. The opposite of belonging is the “absence of the act or will to be with” [16, p.25], and the absence of a feeling of place-belongingness encompasses a sense of loneliness, isolation, alienation and dis-placement [14]. In order to belong, people should be able to express their identity and be recognised as an integral part of their community. The school is an arena that may or may not be inclusive, in the sense that students are able to express their own identity and have the opportunity to develop a sense of belongingness. For example, Rahman [17] explored the relationship between the sense of belongingness to school and the “hidden curriculum” in the literature of Indigenous education. The hidden curriculum includes cultural values, norms and codes associated with school that may be different from students’ cultural backgrounds. The concept, coined by Jackson [18], describes “everything which is not academic, but has important influences on the academic outcomes of the school” [19, p.926]. These are norms and values such as being able to sit quietly and listen to the teacher, wait quietly or cooperate in groups [17]. Rahman [17] expresses that the students must take on another identity that is more academically attuned when attending school.

Few studies have examined positive factors affecting attendance and completion of school. Kearney and Graczyk’s [20] review of interventions to promote school attendance highlighted the importance of collaboration between researchers, educational and mental health professionals, and parents in developing relevant interventions. One example is Rowe and Steward’s [21] work that examined the “whole-school” approach, which aims to promote connectedness to school, a concept that resembles sense of belongingness. In a case study in Queensland, Australia, these authors found that informal teaching, reinforcement and adequate time for relationships to develop were related to school connectedness. Various activities, characterised as positive, social and celebratory that facilitated interaction between school community members, and informal gatherings involving food or events with communal eating, were found to promote school connectedness [21].

Another key gap in existing research is that the adult perspective dominates [20], with few studies focussing on students’ own ideas and aspirations. The active participation of young people is essential to ensure their needs, and contextual factors that shape these, should be addressed [22]. The importance of active participation and an opportunity to provide input is even more important when people come from different backgrounds. Finnmark has the largest proportion of people with Indigenous background within Norwegian regions, specifically the Sámi and the Kveeni [23]. There is no official registration of who has Sámi or Kveeni background in Norway, and because of long time oppression of these Indigenous groups, many are not aware of, or choose to hide their identity. The number of people living in the Finnmark area registered to vote in the Sámi parliament 2013 (being above 18 years) was approximately 8000 [24], which is about 1.1% of the total population of Finnmark [25]. In addition to these Indigenous groups, Finnmark has a number of recent immigrants from countries such as Russia and Finland [26].

To be able to address high dropout rates from school in Finnmark, a shift in focus towards adolescents’ strengths and factors that they perceive as supporting school attendance might lead to novel insights and solutions. Given potentially diverse cultural values and worldviews of different population subgroups, it is vital to have a participatory approach to obtain students’ perspectives and to foster their active involvement in promoting their school environments and health [27].

The aim of this study was to deepen the understanding of factors that support school attendance, as well as possible completion, in a high school in Finnmark, based on the students’ own perspectives. Using a strengths-based approach, we focused on factors that promote attendance, rather than factors that prevent drop out.

## Methods

We used a strengths-based participatory approach with photovoice [28] to enable the creation and sharing of new knowledge through dialogue, with the aim of informing stakeholders for strategies for change [28]. Similar approaches have been used with youth, including Indigenous youth to support participation and empowerment [29,30]. Cooper and Yarbrough [31] reported in their study, combining photovoice and focus groups, that photovoice deepened the discussion and made it easier for the authors to explore health issues in a wider context. In this study, photos generated by the students were used to facilitate dialogue and collaboration with youth through (a) a group conversation, (b) a workshop to thematise the photos and plan a school exhibition, (c) semi-structured individual interviews and (d) the running of an exhibition for a wider audience. The study was notified and recommended by the Norwegian Centre for Research Data (Personvernombudet for forskning). Participation was voluntary and all participants provided informed consent.

### Location and participants

The study was conducted from December 2015 to February 2016 in a high school in a small town in Finnmark (approximately 2250 inhabitants). The municipality has a mixed population of people with Sámi and Kveeni backgrounds, ethnic Norwegians and more recent immigrants. The school, which offers 5 study programmes, has 225 students (2016), most of them aged 16–19 years, with around 40% living away from home.

A strategic selection process was applied to enable a multi-perspective approach [32]. We sought to include participants who could provide insights with respect to the following criteria: (a) gender, (b) age (16 years or older and still in school), (c) study programme (both vocational and theoretical) and (d) living situation (both living away from home and in home). We did not ask for ethnic origin as multiple/mixed ethnic origins are common in the area. Also given the stigmatisation that may be experienced by minoritised ethnic groups, we did not want to force students to specifically identify with a single ethnic identity.

In collaboration with the school’s social worker, a total of seven students were recruited, five girls and two boys. Two were in vocational study programmes while the other five attended theoretical programmes. All seven were in second or third grade, meaning that they were older than 16 years. Three of them lived with their parents, and four lived away from home.

### Data collection

In a preparatory meeting with the seven students in their school in December 2015, the first author described the overall project purpose and asked them to use their cell phones to take two to five photographs that illustrated why they liked being in school or why they wanted to complete their high school education. They were encouraged to base their choice of photos on their own sense of what was significant, either in school, at home or in other places where they spent leisure time. The students were free to ask any questions during this meeting, regarding the project or their assigned task. Through the five-week process, the first author was available to be contacted, and facilitated the process by sending reminders via SMS and cell phone calls. No technical problems related to the process of taking photos were reported.

The second visit to the school lasted 10 days in February 2016. After a first meeting to provide students a time schedule of the planned activities (i.e. methods), data generation steps included the following.

#### Group meeting to jointly explore the content of the photos

The first author met the seven students together to jointly explore and discuss the photos. In total, 21 photos were submitted by the students, all printed in A4-format. The number of photos per student varied, from two to four. Diverse motives for taking the photos were shared when students described the thoughts related to their photos. Further, there were group discussions related to each photo, where the other students, as well as the researcher were free to ask questions, provide comments and share own related reflections. The meeting lasted about 1.5 hours.

#### Workshop to thematise photos and plan an exhibition

Two days after the initial group conversation, and as part of the participatory approach of the study, all seven students attended a workshop to thematise the photos. They were encouraged to generate themes reflecting the meaning of photos, and assign photos to the different themes. The six themes students generated were friendship, academic encouragement, break (during school), good environments (both physical and social), recuperation (outside of school), and goals and ambitions. During this work, and based on the participatory action approach of the study, the first author introduced the idea of making a photo exhibition in the school canteen to inform students, teachers as well as other stakeholders of their issues of interest and concern. The students responded positively to this proposal, and they were also supportive of the idea of inviting the local newspaper to write about the project. They created titles for photos or provided small descriptions in writing to be used in the exhibition. The plan for an exhibition was only introduced at this stage of the study after gauging the group’s enthusiasm and energy for such an endeavour, and also to avoid creating stress at the beginning of the process.

#### Individual interviews

To ensure an in-depth understanding of students’ experiences and reflections regarding school attendance, all students were further invited to participate in a follow-up individual interview. This enabled students to address issues they may not have wanted to discuss in plenum, or that may have arisen from ongoing reflection following the group discussion. Individual semi-structured interviews were subsequently conducted with four students who chose to participate in this stage. The interviews took place in a room in the school, and varied between 20 and 30 minutes.

#### School exhibition

Based on the action-oriented approach of the study which encompassed informing policy-makers and stakeholders of the issues of concern, the students hosted an exhibition of their photos in the school canteen. This occurred on the last day of the researcher’s visit to the school. The local newspaper was invited and published a news report based on interviews with two students and the first author. A few days later, the school administration presented the exhibition during a parental meeting, and information about the study was also posted on the school homepage and Facebook pages. The exhibition is still present in the canteen, nearly 2 years following study completion.

#### Participant observation

During the field visits, the first author participated in student activities, such as meals, physical training activities and national Sámi day, during which the whole school participated. Approximately 25% of the students were wearing traditional Sámi national clothing, including one of the study participants. These participatory activities were aimed at creating a wider contextual understanding to inform subsequent analysis.

### Data analysis

All group conversations and individual interviews were audio-recorded and transcribed verbatim, except for the conversations during planning and running of the school exhibition. The data were analysed by qualitative content analysis inspired by Graneheim and Lundman [33]. First, meaning units were created based on the raw data. Further, the meaning units were condensed into codes. Codes were then abstracted and aggregated to sub-categories that finally were grouped into categories. The first, second and last authors were involved in this process due to language familiarity. Quotations were translated from Norwegian to English by the first author. The names allied to the quotations are fictional. Table 1 illustrates the data analysis process. When comparing the categories that emerged from the content analysis with the themes that the students generated for the exhibition, we found a high degree of correspondence.

**Table 1.** Example of analysis from meaning condensation to category.

Quotations	Meaning condensation	Code	Sub-category	Category
This is from the breaks and the tea table with friends. You need the breaks in school to get you through the day. There is such a tea table in every building, so that is very ok for us	Here is a picture from a break, the tea table and friends. You need a break to get through the day at school. The tea table is ok for us	Pauses and breaks with friends is important during the day in school	Pauses and breaks during the school day	Recuperation and recreation
I have a picture from a concert. And the week before that we had lots of school, and then it is ok to get a reward. And when I carry out everything I can take a small reward. And it also represents a small motivation so that you know that you can do something that you appreciate when you have done some good work, like getting a good grade	I have a picture from a concert. During the week before we had lots of school, so then it was good to get a reward. To do something you appreciate represents a motivation for doing some good work, like getting a good grade	Rewarding oneself with something one appreciates after doing some good work is motivating	Recuperation and rewards outside of school	

## Results

**Figure 1.**
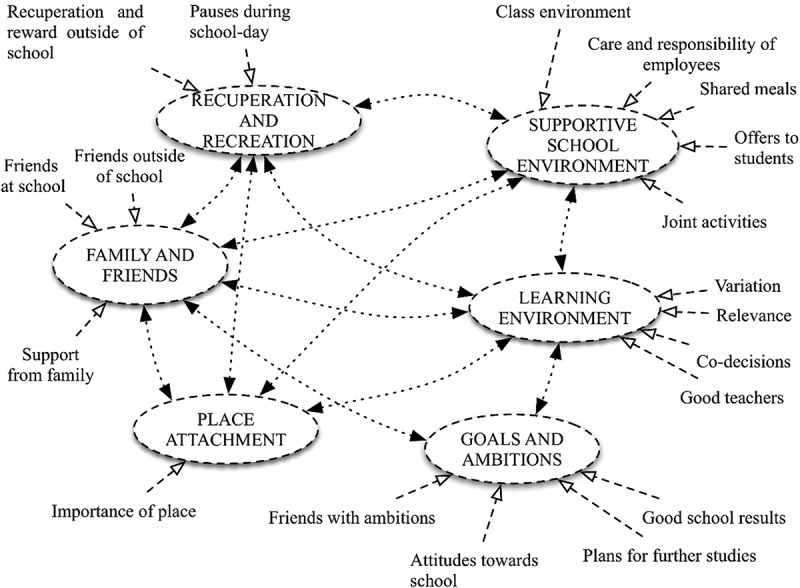
A network of school attendance factors.

Six categories were identified as describing attendance-factors in high school: Supportive school environment, good learning environment, family and friends, recuperation and recreation, goals and ambitions, and place attachment. As illustrated in Figure 1, the categories were highly interrelated.

### Supportive school environment

A socially supportive school environment was of significance for the students’ well-being, thriving and school attendance. The students experienced the employees at school as both caring and responsible, and the way the teachers and students interacted was relatively informal. These ways of relating were highlighted as important for their well-being. When they felt a need for talking to an adult, there was always “a door open”. Siri said: “You can always come and talk to a teacher even if they are not teaching in your class. And most certainly to the principal and head of unit in your line of study”. They also valued the teachers’ serious and immediate response to bullying or peer victimisation. One of the students expressed that he felt the teachers sensed if anything was wrong, and took the students aside to talk to them if needed. Another aspect noted was the school’s policy of contacting those who did not turn up at school in the morning, to check on them. The students reflected on the sense of being taken care of as especially important for those who were not particularly emotionally strong. They appreciated that the school collaborated closely with their parents, and, in particular, those who lived away from home, expressed that they felt they were taken care of by the school social worker.

The students appreciated being treated not only as students, but also as whole human beings by their teachers. Siri said: “They do not have to be your best friend, but show that they are not just doing their job for the money, that they can offer something of themselves as well”. Several of the students said that the social environment in the class was a key factor for wellbeing at school. Good dynamics in the class created a humorous atmosphere. This again seems to have a committing function that created a sense of belonging, meaning that it helped the students to view themselves as part of a larger whole, for which they shared a responsibility. This was described by Kari as follows: “We have an arrangement that if someone does not show up one day, we will call or send a message to check if he or she is ill or if they have just over-slept”. As also explained by Siri: “We have a great class environment, and really enjoy being together. You feel a bit empty if you have not been to school one day because they always put you in a good mood”.

**Figure 2.**
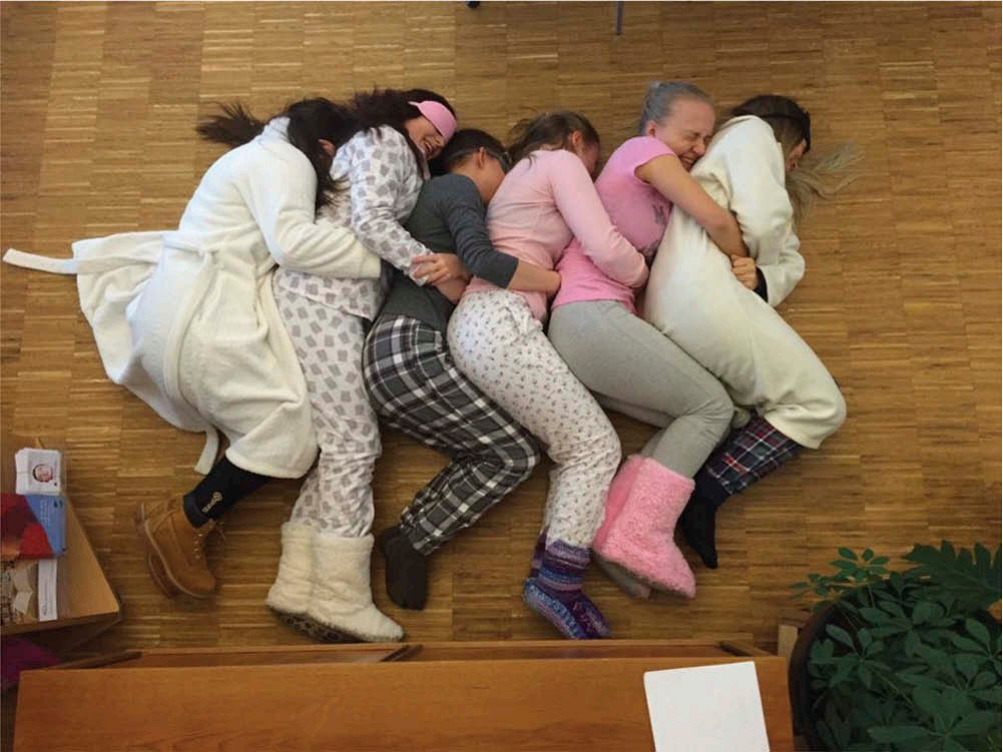
From a pyjamas-day.

Several students had taken photos of different kinds of school-related social activities, such as teacher-organised activities outside of the school property, for instance, a biology class by the riverside and a volleyball tournament on the beach. These illustrated the perceived importance of bringing students together across class boundaries, so that they learned to know each other in different kinds of settings and felt more relaxed with many of the other students. Figure 2 illustrates a student-organised activity, where they were allowed to use the schools’ premises outside of school hours for a gathering. Nora explained:

Here we had a theme-day with the final-year students. It was pyjamas-day, and that was a very good theme. And then it´s really cozy that all the final-year students come together, and then we take pictures of each other. These are events to look forward to if daily life at school is a bit boring.

Almost every day the school offers leisure activity for the students, which seemed particularly important for students who live away from home. Anna said: “The school has been good in making things work for the students who live away from home, through the free breakfast, the Tuesday evening arrangement where everyone can meet, and the study café on Wednesdays”.

**Figure 3.**
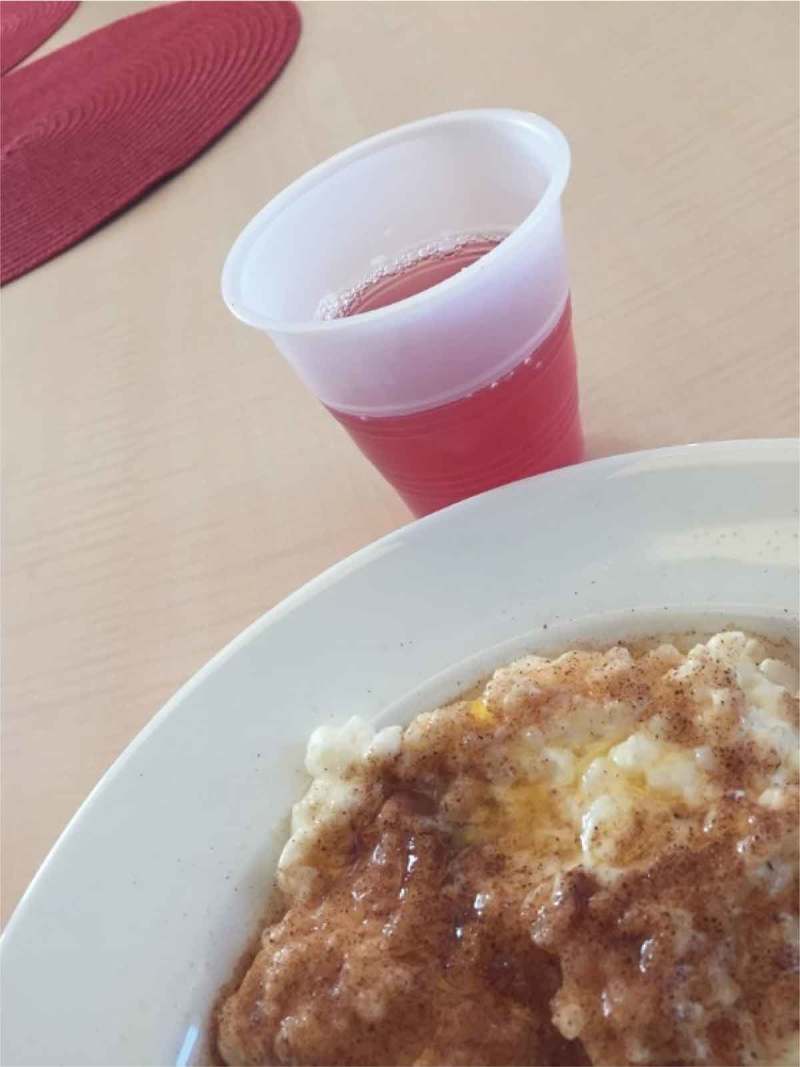
Free porridge for lunch once a week.

The school meals played an important role for all of the students, both for the meal itself and because the meals functioned as a social meeting place. Susanne said: “To eat breakfast and lunch with Ida is pretty nice. It makes me thrive. And the breakfast is free as well, and that is really good. It also represents a motivation for coming to school” (Figure 3). Based on observations while visiting the school, the first author noted that some of the teachers also had breakfast at school and that this seemed to be a valuable social arena among students and teachers as well.

### Good learning environment

In addition to emphasising the importance of having good relationships with teachers, the students had high regards for a varied approach to education, and appreciated having a day at school that was more than sitting at a desk listening to teachers. Didactical variation, including small breaks, was well received by the students, including Kristian who explained:

For us, who have a lot of writing assignments, and do a lot of sitting, it has been important that we can have fun with each other, or that the teachers pay attention to the fact that we have to do something that is amusing once in a while. Because that will strengthen the class environment.

They also appreciated the flexibility of the teachers when it came to ways of learning. One of them expressed that it was important for her to listen to music while working, and thus she very much appreciated being allowed to wear earphones in class.

A participatory approach to education, where the students could have a say in terms of what happened in the classroom, was valued. The students appreciated pedagogical approaches that were adapted to the specifics of subject matter because they felt that this made the time in school more enjoyable and also supported their gradual mastering of the subject being taught. Anders was happy about the specific approach to his study area:

We are occupied with vehicles, and then it does not make any sense to sit and write the whole day like the students in theoretical programs. You do not learn how to fix an engine by reading a book. It might help, but you do not learn it.

### Recuperation and recreation

**Figure 4.**
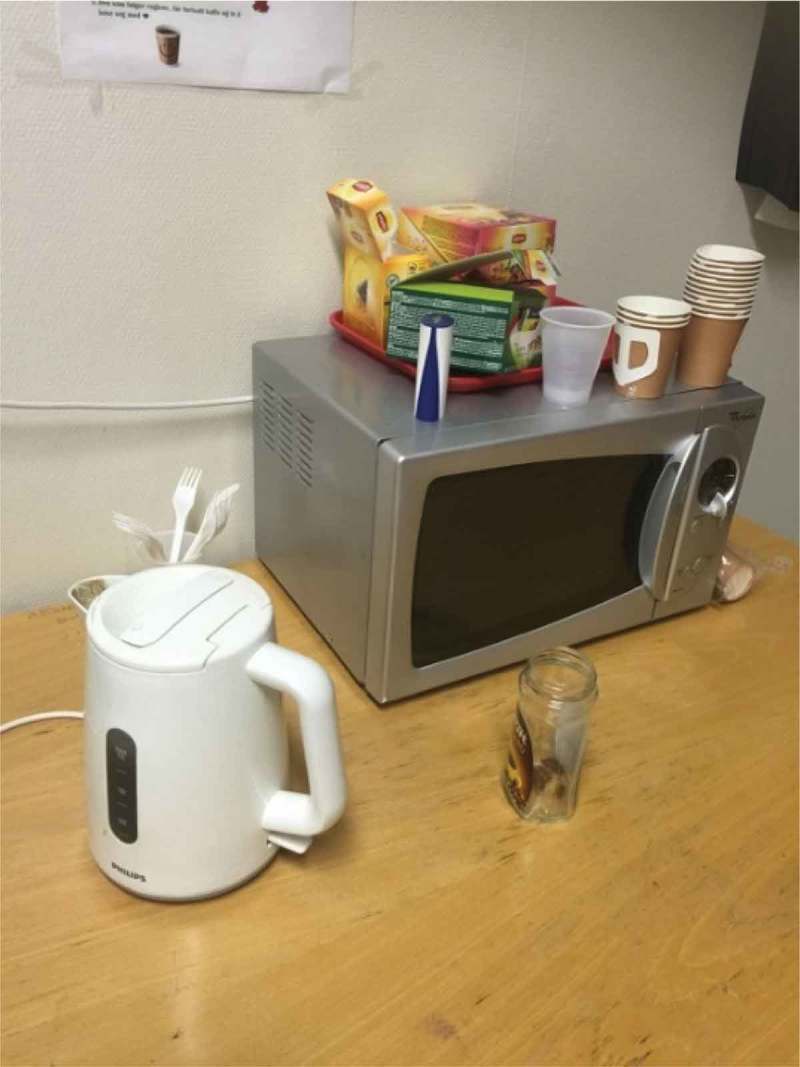
The tea corner.

**Figure 5.**
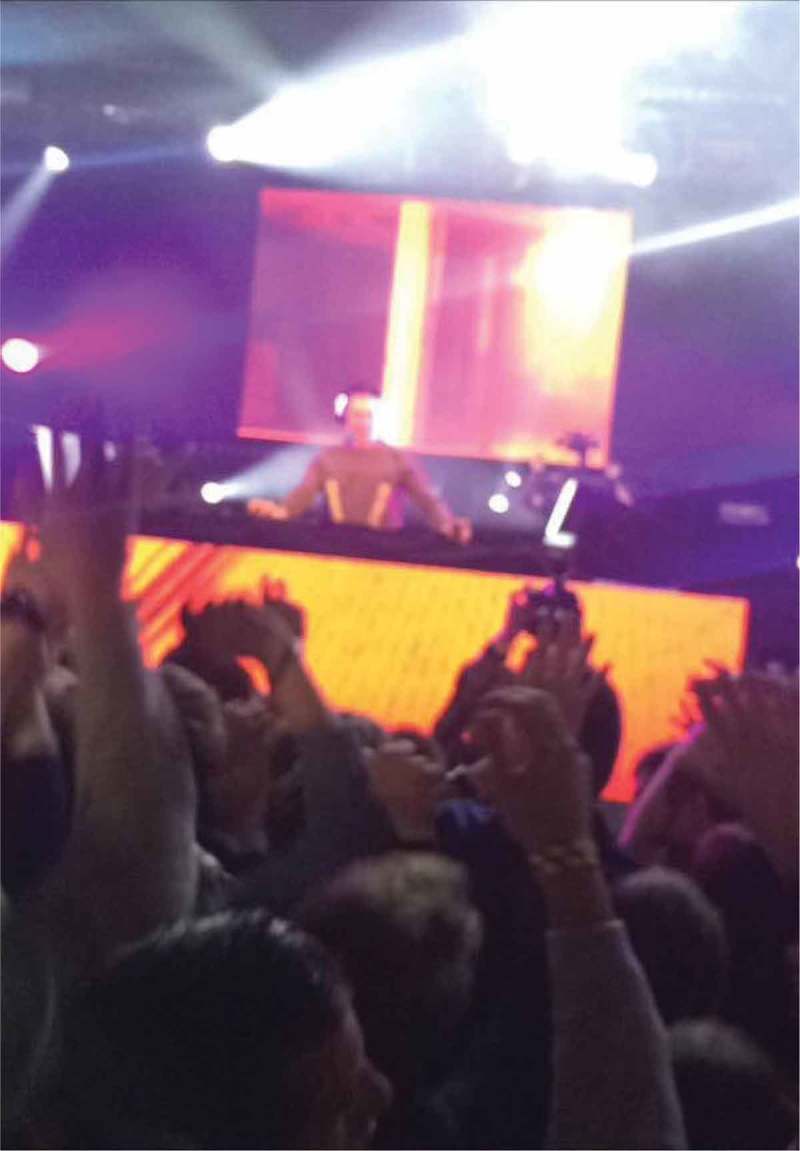
From a concert. An example of outside of school recuperation.

The need for recuperation and recreation was clearly expressed during both group and individual conversations. The students described the importance of a balance between school and time off, and their need for breaks both in and outside of school. According to Anna: “You need breaks during the day at school to get you through the day”. During the breaks, the students would spend their time informally socialising with other students, often in connection with eating or drinking. A kettle and a coffee machine were available in a small corner of a room for student use, which they called the “tea corner” (Figure 4) and highlighted as an important gathering place. During ordinary school days, students could look forward to breaks outside of school, which were regarded as rewards and opportunities to reconstruct individual integrity and express identity (Figure 5).

### Family and friends

Having good friends was important for the students, and the school supported friendship by organising social activities both within and outside the class. Kari expressed the importance of friends as a part of the school environment:

I believe that what happens around the school has something to say, that you have someone to spend your leisure time with, that you are not alone. Because when you have friends they can for example push you to go to school a day when you do not feel like going

**Figure 6.**
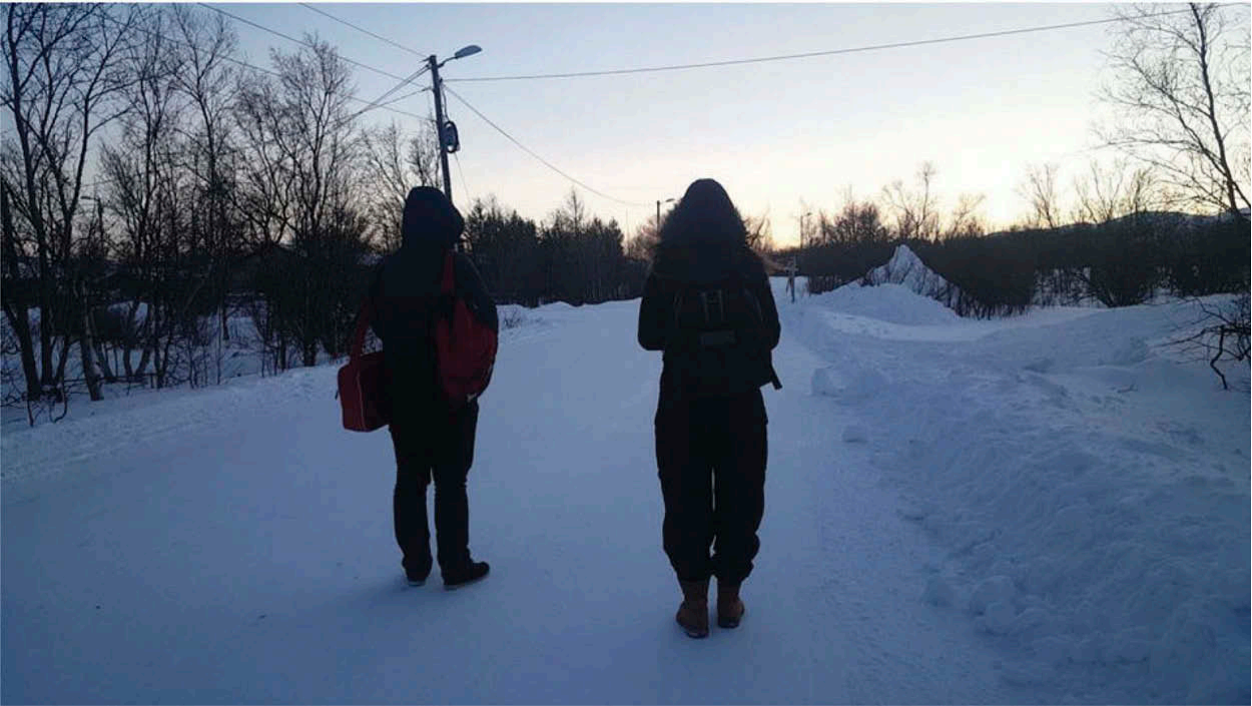
Together with friends – home from school.

Figure 6 illustrates the significance of the school as an arena for making friendships, as expressed Kari:

Here I am on my way home from school. The thing is that I think it is a bit cozy when one is done with the day at school, and just asks: ”Is anyone going up towards Heather Road?”, and then there are always two more that are heading in that direction, and then we just team up.

A close connection with parents was also highlighted as important for well-being. Explicit support from home was said to be especially important for the students who lived away from home. Anders said: “That you have parents who care is also important. Especially for those who live away from home, that they pay attention even if you do not live at home”.

### Goals and ambitions

Many of the students were interested in performing well at school and getting good grades. Receiving good grades supported their feeling of mastery, especially when they knew that they had worked hard and seriously to achieve them. Nora expressed her satisfaction in getting a good grade by saying:

The point is that I received an A. This was an assignment that I actually thought was enjoyable and I had the opportunity to write about something that really interested me. I did some good work on it and then I was rewarded for the job, and that felt good.

Having goals for the future represented a strong motivation for attending school. In thinking about high school as a stepping stone towards her future desired career, Siri expressed:

The most important part of going to school is that there is a higher education in the other end of it. I could end up with the education that I am really interested in. If I get good grades I will be able to enter the study program I really want to be part of … and then I will have the life I want in a sense.

For the students, it can be easier to talk to friends and peers about their plans for the future, because they have more in common. According to Kristian, the conversations with friends represents a multi-faceted dialogue that contains not only information but also inspiration and engagement regarding the future:

Another thing lately is that friends can represent a small piece of information when we talk about the future, because we have the same position in life. It is not like you only get some advice, but more that you become much more engaged when you talk to friends about what they would like to become and the possible study options.

### Place attachment

The actual location of the school emerged as important for the students’ thriving. Many of them described with enthusiasm different areas in the school surroundings that they used for recreation during leisure time. Kari said: “Thus, I like…. (The place) is really such a nice place (…). There is no such bad environment, or so”. Anders conveyed a strong sense of identity connected to the place by saying he could never have gone to school in another part of the country: “I should have taken a photo of the county in itself. I would never have managed to go to school in Oslo, I cannot stand the big city. I’m going crazy”. Even if they had different opinions about seasonal darkness, this also seems to be a strong part of their identity, as it was described in pictures and discussed with great involvement.

## Discussion

An important finding of the present study is the identified related nature of supportive environment and belongingness, where teachers and other school staff made important contributions to creating and promoting both a supportive social and educational environment. The importance of having social support from the environment was expressed in the students’ narrations and photos in different ways and seemed to reinforce a sense of being a part of something greater. These results are in line with previous cross-sectional and longitudinal studies examining the relation between teacher and peer social support and school engagement, connectedness to school and self-reported positive health [34–36]. A supportive environment seems also to have impacted on the students supporting each other, highlighting the importance of the school’s influence on processes of friendship.

What is important to note in the present study is that nearly 40% of its students are away from home during the semesters. They are quite young, between 16 and 19 years, and have to handle a lot on their own. Based on these circumstances, the school environment and support from classmates and school staff appeared to be even more important than for students who can return home to their parents in the afternoon and have their friends around. The students described how free breakfast and evening arrangements as well as other kinds of school related social activities were significant for well-being and a motivation to attend school. Through these arrangements, school staff may have increased the feeling of being “at home” in school, that is of being comfortable, secure and emotionally attached. Such a feeling is related to a sense of belongingness in general [14,15]. The students came from different parts of the county and had different cultural backgrounds, but a whole school ethos or culture seems to have been created. In this culture, the students were able to express their own identity and were recognised for who they were, contributing to sense of belonging [17,18], a factor shown in other studies to promote school attendance and hinder dropout [21].

The variety of opportunities for social activities described in this study, and the common experience of the students of being taken care of by the school leaders, reflected a widespread collaboration between different sectors and professions, such as the teachers, social workers and the school administration. Similar social factors were also identified as important in other studies [20].

The educational environment seemed to support the students’ sense of participating in a field of knowledge. The students expressed valuing an approach to learning where human, social and democratic dimensions contextualise the subject matter. The reason for this seems to be strongly related to the socially supportive school environment and the school leaders’ ability to see the students as “whole persons”, as expressed by the students themselves. The students also conveyed that they were allowed to express their ideas and aspirations in the school setting. They appreciated the ability to participate and have a say in terms of what happened in the classroom and expressed how this supported their attachment to school. Other studies have found that teachers who enabled and supported independence among their students could contribute to a higher completion rate [34].

The students expressed the importance of breaks and other opportunities to recuperate during the school day and outside school. They valued pedagogical variation, which is in accordance with the results by Warne and colleagues’ [37], who found that a good learning environment includes a shift between play and work. In their study, which was also a photovoice study, recuperation was said to “recharge batteries” and be important for the continuation of school work. The breaks made it possible for students to do what was important to them, and was said to reconstruct individual integrity. In agreement with Warne and colleagues [37], we found that recuperation is important not only during weekends, holidays and after-school, but also during the school day, enabling the ability to grow friendship, be rewarded and have the opportunity to express identity .

### Methodological strengths and limitations

The use of photovoice supported dialogue, collaboration and active participation of students, which strengthened the depth of data generated. We observed that the students worked independently in the generation of motives, and that the related group discussion and follow-up individual interviews contributed to valuable insight into their diverse life worlds that would not have been generated otherwise. Such an approach better mitigated any bias stemming from researchers’ expectations than other less participatory methods would have. Although data collection was conducted by the first author, analysis and dissemination processes were accomplished in close collaboration among the authors enabling multiple perspectives to strengthen these processes.

Although the present study was inspired by participatory action research, the scope of the study did not allow for action beyond mobilising information back to the teachers, school administration and local community (via the local newspaper), as part of the school exhibition. The scope of the present study was also geographically limited, with participants being drawn from a single school. In this study, we focused on factors that can promote school attendance. Although this was intended, such a focus may have influenced the interpretation of the results by not problematising issues of concern. By including additional schools in the study, or the students’ personal experiences of problems and contradictions related to attendance, more insight could have been gained. We recommend such limitations be considered in the design of future research on this topic.

## Conclusion

Few studies have examined broader economic, political and social factors shaping school dropout [13]. The present study illustrates the need for broadening the focus from an individual level to a higher level. Listening to the voices of the students, it has become clear that they emphasise the importance of the school environment, socially and spatially, when asked what promotes school attendance. In recognising the school-internal and school-external attendance factors as an interactive network, the school and policy-makers can become facilitators of attendance. Such an approach necessitates the need for an open, boundary-free approach, where all relevant factors are considered, in agreement with what Green and colleagues [38] describe as settings that can promote health. The classical role of the school as a unit for knowledge transfer must continuously be re-constructed, and the boundaries between school and its environment must become increasingly permeable. Competencies other than mere didactic ones must be incorporated into school relationships and activities in order to strengthen factors that support attendance and, in the long-term, enable health and well-being in coming generations.

## References

[1] Albert C, Davia MA. Education is a key determinant of health in Europe: a comparative analysis of 11 countries. Health Promot Int. 2011;26(2):163–11. 20935091 10.1093/heapro/daq059

[2] Dahl E, Bergli H, Wel KAVD. Sosial ulikhet i helse: en norsk kunnskapsoversikt. Oslo: Oslo and Akershus University College of Applied Sciences: Oslo and Akershus University College of Applied Sciences; 2014.

[3] Marmot M The status syndrome: how social standing affects our health and longevity. New York (NY): Henry Holt and company; 2005.

[4] Completion rates of pupils in upper secondary education, 2010-2015 [Internet]. 2016 Available from: https://www.ssb.no/en/utdanning/statistikker/vgogjen/aar/2016-06-02.

[5] Government.no Increasing completion rates at upper secondary schools. Oslo: Ministry of Education and Research; 2015; Available from https://www.regjeringen.no/en/topics/education/school/innsiktsartikler/program-for-bedre-gjennomforing-i-videregaende-opplaring/id2005356/.

[6] Willms JD Student engagement at school. A sense of belonging and participation. Results from Pisa 2000 In: DEVELOPMENT OOFEC-OA, editor. OECD: ORGANISATION FOR ECONOMIC CO-OPERATION AND DEVELOPMENT. 2003.

[7] Fredricks JA, Blumenfeld PC, Paris AH School engagement: potential of the concept, state of the evidence. Rev Educ Res. 2004;74(1):59–109.

[8] Archambault I, Janosz M, Fallu J-S, et al Student engagement and its relationship with early high school dropout. J Adolesc. 2009 6 1;32(3):651–670. 18708246 10.1016/j.adolescence.2008.06.007

[9] Froiland JM Parents’ weekly descriptions of autonomy supportive communication: promoting children’s motivation to learn and positive emotions [Article]. J Child Fam Stud. 2015 1;24(1):117–126. PubMed PMID: WOS:000347528700011; English.

[10] Green CL, Walker JMT, Hoover-Dempsey KV, et al Parents’ motivations for involvement in children’s education: an empirical test of a theoretical model of parental involvement. J Educ Psychol. 2007;99(3):532–544.

[11] Markussen E, Lødding B, Holen S De’ hær e’kke nokka for mæ. Om bortvalg, gjennomføring og kompetanseoppnåelse i videregående skole i Finnmark skoleåret 2010-2011 In: NIFU, editor. NIFU report. Vadsø: NIFU; 2012.

[12] McNeely CA, Nonnemaker JM, Blum RW Promoting school connectedness: evidence from the national longitudinal study of adolescent health. J Sch Health. 2002;72(4):138–146. 12029810 10.1111/j.1746-1561.2002.tb06533.x

[13] De Witte K, Cabus S, Thyssen G, et al A critical review of the literature on school dropout. Educ Res Rev. 2013;10:13–28.

[14] Antonsich M Searching for belonging – an analytical framework. Geography Compass. 2010;4(6):644–659.

[15] Yuval-Davis N Belonging and the politics of belonging. Patterns Prejudice. 2006 7 1;40(3):197–214

[16] Sicakkan HG, Lithman YG Politics of identity, modes of belonging and citizenship: an overview of conceptual and theoretical challenges In: lithman YG, Sicakkan HG, editors. Changing the basis of citizenship in the modern state: political theory and the politics of diversity. Lewiston: N.Y Edwin Mellen Press; 2005 p. 1–35.

[17] Rahman K Belonging and learning to belong in school: the implications of the hidden curriculum for indigenous students. Discourse: Stud Cult Politics Educ. 2013 12 1;34(5):660–672.

[18] Jackson PW Life in classrooms. New York (NY): Holt, Rinehart and Winston; 1968 English.

[19] Sari M, Doganay A Hidden curriculum on gaining the value of respect for human dignity: a qualitative study in two elementary schools in adana. Educ Sciences: theory Pract. 2009;9(2):925–940.

[20] Kearney CA, Graczyk PA Response to intervention model to promote school attendance and decrease school absenteeism [journal article]. Child Youth Care Forum. 2014 2 01;43(1):1–25.

[21] Rowe F, Stewart D Promoting connectedness through whole-school approaches. Key elements and pathways of influence. Health Educ. 2011;111(1):49–65.

[22] Woodman D, Wyn J Youth policy and generations: why youth policy needs to ‘Rethink Youth’. Soc Policy Soc. 2012;12(2):265–275.

[23] Verrill E The Kveeni of Northern Norway: from national minority to indigenous people: Brandeis Institutional Repository. Waltham, MA: The Faculty of the Graduate School of Arts and Sciences, Brandeis University; 2014.

[24] Slaastad TI Samisk statistikk 2016. Sámi statistihkka 2016 In: Statistics-Norway, editor. Reports. Oslo: Statistics Norway; 2016.

[25] Statistics-Norway Folkemengde og befolkningsendringar, 1. januar 2016 Oslo. 2016 Available from: https://www.ssb.no/befolkning/statistikker/folkemengde/aar-per-1-januar/2016-02-19?fane=tabell&sort=nummer&tabell=256001

[26] Høydahl E Innvandrere i bygd og by In: Samfunnsspeilet 2/2013. Oslo: Statistics Norway; 2013.

[27] Castagno AE, Brayboy BMJ Culturally responsive schooling for indigenous youth: a review of the literature. Rev Educ Res. 2008;78(4):941–993.

[28] Wang C, Burris MA Photovoice: concept, methodology, and use for participatory needs assessment. Health Educ Behav. 1997;24(3):369–387. PubMed PMID: 9158980. 9158980 10.1177/109019819702400309

[29] Delgado M Urban youth and photovoice: visual ethnography in action. Oxford: Oxford University Press; 2015.

[30] Castleden H, Garvin T Modifying Photovoice for community-based participatory Indigenous research. Soc Sci Med. 2008 3;66(6):1393–1405. PubMed PMID: 18191883; eng. 18191883 10.1016/j.socscimed.2007.11.030

[31] Cooper CM, Yarbrough SP Tell me—show me: using combined focus group and photovoice methods to gain understanding of health issues in rural guatemala. Qual Health Res. 2010;20(5):644–653. PubMed PMID: 20154296. 20154296 10.1177/1049732310361894

[32] Silverman D Doing qualitative research. 4th ed. Los Angeles: Sage publications; 2013.

[33] Graneheim UH, Lundman B Qualitative content analysis in nursing research: concepts, procedures and measures to achieve trustworthiness. Nurse Educ Today. 2004 2 01;24(2):105–112. 14769454 10.1016/j.nedt.2003.10.001

[34] Hardre PL, Reeve J A motivational model of rural students’ intentions to persist in, versus drop out of, high school. J Educ Psychol. 2003;95(2):347–356.

[35] Wang M-T, Eccles JS Social support matters: longitudinal effects of social support on three dimensions of school engagement from middle to high school. Child Dev. 2012;83(3):877–895. 22506836 10.1111/j.1467-8624.2012.01745.x

[36] Warne M, Snyder K, Gillander Gadin K Participation and support - associations with Swedish pupils’ positive health. Int J Circumpolar Health. 2017;76(1):1373579 PubMed PMID: 28911274; eng. 28911274 10.1080/22423982.2017.1373579 PMC5645769

[37] Warne M, Snyder K, Gillander Gadin K Promoting an equal and healthy environment: swedish students’ views of daily life at school. Qual Health Res. 2013 10;23(10):1354–1368. PubMed PMID: 24062421; eng. 24062421 10.1177/1049732313505914

[38] Green LW, Poland BD, Rootman I The setting aproach to health promotion In: Poland BD, Green LW, Rootman I, editors. Settings for health promotion: linking theory and practice. Thousand oaks (CA): Sage publications; 2000 p. 1–43.

